# Global, cancer-specific microRNA cluster hypomethylation was functionally associated with the development of non-B non-C hepatocellular carcinoma

**DOI:** 10.1186/s12943-016-0514-6

**Published:** 2016-04-30

**Authors:** Masanori Nojima, Takeshi Matsui, Akihiro Tamori, Shoji Kubo, Ken Shirabe, Koichi Kimura, Mitsuo Shimada, Tohru Utsunomiya, Yasuteru Kondo, Etsuko Iio, Yutaka Naito, Takahiro Ochiya, Yasuhito Tanaka

**Affiliations:** Institute of Medical Science Hospital, Center for Translational Research, the University of Tokyo, Tokyo, Japan; Department of Gastroenterology, Teine Keijinkai Hospital, Sapporo, Japan; Department of Virology and Liver Unit, Nagoya City University Graduate School of Medical Sciences, Nagoya, Japan; Department of Hepatology, Osaka City University Graduate School of Medicine, Osaka, Japan; Department of Hepato-Biliary-Pancreatic Surgery, Osaka City University Graduate School of Medicine, Osaka, Japan; Department of Surgery and Science, Graduate School of Medical Sciences, Kyushu University, Fukuoka, Japan; Department of Surgery, Tokushima University, Tokushima, Japan; Division of Gastroenterology, Tohoku University Hospital, Sendai, Japan; Division of Molecular and Cellular Medicine, National Cancer Center Research Institute, Tokyo, Japan

**Keywords:** Global hypomethylation, MicroRNA, MicroRNA cluster, MicroRNA regulation, Statistical modeling, Non-B non-C hepatocellular carcinoma

## Abstract

**Background:**

While hepatitis B and C viral infection have been suppressed, non-B non-C hepatocellular carcinoma (NBNC-HCC) is considered to be rising in incidence terms in some developed countries where prevalence of those viral infections among HCC patients had been very high (such as Japan, Korea, and Italy). To elucidate critical molecular changes in NBNC-HCC, we integrated three large datasets relating to comprehensive array-based analysis of genome-wide DNA methylation (*N* = 43 pairs) and mRNA/miRNA expression (*N* = 15, and 24 pairs, respectively) via statistical modeling.

**Results:**

Hierarchical clustering of DNA methylation in miRNA coding regions clearly distinguished NBNC-HCC tissue samples from relevant background tissues, revealing a remarkable tumor-specific hypomethylation cluster. In addition, miRNA clusters were extremely hypomethylated in tumor samples (median methylation change for non-clustered miRNAs: -2.3%, clustered miRNAs: -24.6%). The proportion of CpGs hypomethylated in more than 90% of the samples was 55.9% of all CpGs within miRNA clusters, and the peak methylation level was drastically shifted from 84% to 39%. Following statistical adjustment, the difference in methylation levels within miRNA coding regions was positively associated with their expression change. Receiver operating characteristic (ROC) analysis revealed a great discriminatory ability in respect to cluster-miRNA methylation. Moreover, miRNA methylation change was negatively correlated with corresponding target gene expression amongst conserved and highly matched miRNA sites.

**Conclusions:**

We observed a drastic negative shift of methylation levels in miRNA cluster regions. Changes in methylation status of miRNAs were more indicative of target gene expression and pathological diagnosis than respective miRNA expression changes, suggesting the importance of genome-wide miRNA methylation for tumor development. Our study dynamically summarized global miRNA hypomethylation and its genome-wide scale consequence in NBNC-HCC.

**Electronic supplementary material:**

The online version of this article (doi:10.1186/s12943-016-0514-6) contains supplementary material, which is available to authorized users.

## Background

Hepatocellular carcinoma (HCC) is a common refractory cancer especially in areas where hepatitis virus infection is common, including Japan. (746,000 deaths/782,000 new cases in the world, GLOBOCAN 2012) [1]. While hepatitis B virus (HBV) and C virus (HCV) infection have been suppressed with development of public health measures and medical treatment, non-B non-C hepatocellular carcinoma (NBNC-HCC) is rising in incidence terms in Japan [2–4]. The relative proportion of NBNC-HCC in respect to total HCC has been increasing in etiological terms from approximately 10% to 15-20%, with elevated rates of nonalcoholic fatty liver disease (NAFLD) also being a potentially contributory factor [2–4]. A similar tendency is observed in some developed countries where prevalence of HBV or HCV infection among HCC patients had been very high (around 80-90%), such as Korea, and Italy [5]. The mechanism(s) underlying the carcinogenesis of NBNC-HCC can be different from virus-mediated cancers; accordingly, to suppress all types of liver cancer efficiently, the molecular basis of each etiology should be elucidated.

In terms of molecular oncology, altered epigenetic regulation is known to be an important contributory factor in tumor development. Epigenetic marks such as DNA methylation patterns are observed to be significantly different between cancer and non-cancer tissues, and a number of studies have indicated various cancer-specific epigenetic characteristics for every cancer type [6, 7]. In terms of HCC, altered DNA methylation patterns are also indicative of tumor-associated processes [8–11]. The main focus of DNA methylation research has been on cancer-specific CpG island methylation within promoter regions of individual genes of interest, but the recent development of epigenome-wide analysis approaches has enabled the DNA methylome to be assessed comprehensively [9, 11, 12]. Genome-wide hypomethylation is a common epigenetic change in tumor cells compared to normal cells, as well as region-specific hypermethylation of promoter regions of cancer-related genes [13]. Recently, it was reported that HBV encodes a DNA methylation suppressor protein (HBx). This factor is considered oncogenic, with a strong influence on genome-wide methylation experimentally revealed [12, 14, 15]. Whether there are external “epimutagens” in other tumors including NBNC-HCC is an important research question.

To study the dynamic interplay between DNA methylation and messenger RNA (mRNA) expression, epigenetic information should be connected to genome-wide expression information; however, such integration is a challenging task. Even if genome-wide surveillance is employed, the full extent of the generated data is not often completely utilized or appreciated. In addition, gene activity is also controlled post-transcriptionally by microRNAs (miRNA), with this complexity making the regulation of general (not local) gene expression more difficult to understand [16]. Oncogenic miRNAs have been reported, as well as oncogenic epigenetic alterations, in the case of HCC [11, 17]. For example, expression of has-mir-216a and b was found to be upregulated in HCC, with oncogenic behavior also observed for both miRNAs [17]. miRNA expression can also be regulated by DNA methylation, albeit the correlation between these is not always positive or negative and is region-dependent [18]. Moreover, although DNA methylation alterations in CpG islands within promoter regions have been well studied in the oncology filed, the dynamics and function(s) of DNA methylation in other regions, such as gene bodies or non-island CpG sites, is poorly understood [19].

Here, in this study, to elucidate critical molecular changes in NBNC-HCC, we integrated three large-scale datasets relating to comprehensive analysis of genome-wide DNA methylation and mRNA/miRNA expression, together with statistical modeling. We firstly focused on comparison of genome-wide DNA methylome and mRNA transcriptome between tumor and non-tumor background tissues. After detecting tumor-specific differences in the DNA methylome, we explored the consequences of these alterations at a functional level in terms of corresponding expression changes, if present. We also found a remarkably broad pattern of aberrant DNA methylation within miRNA coding regions, with its relationship to target gene expression determined by constructing statistical models. Our finding describes the dynamics of DNA methylation-expression interplay and associated modification by concomitant epigenetic alteration of miRNAs. We believe that this study provides highly generalizable findings relating to global regulation of gene expression as influenced by epigenetic alterations during carcinogenesis.

## Results

### Differences in methylome state between NBNC-HCC tumor tissues and non-tumor background tissues

**Fig. 1:**
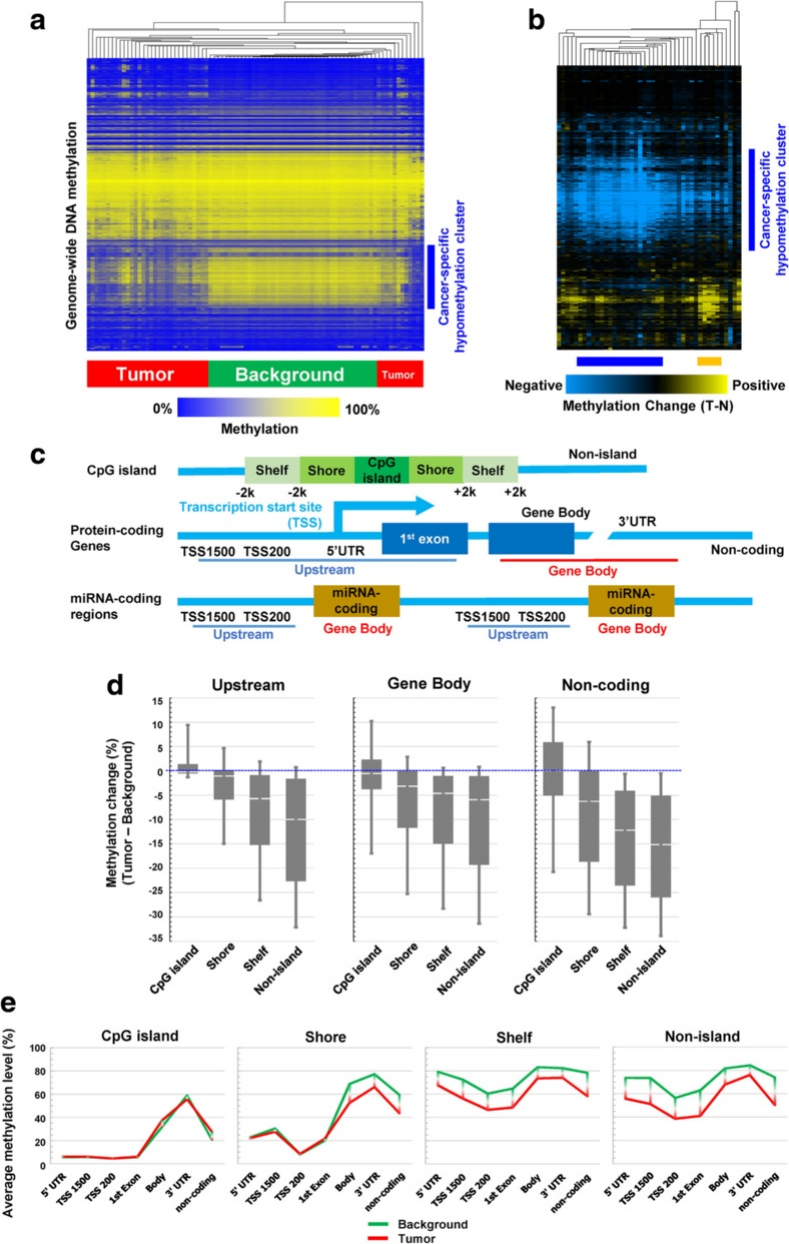
Genome-wide DNA methylation analyses of tumor and background tissues in non-B non-C hepatocellular carcinoma patients. **a**. Hierarchical clustering of genome-wide methylation data from analysis of tumor and background tissues. Each column corresponds to a single sample. Methylation analysis was performed using the Infinium HumanMethylation450 BeadChip. One percent of probes were randomly sampled for the analysis. **b**. Hierarchical clustering of the difference in genome-wide methylation level between tumor and background pair tissues. Each single column corresponds to each case. Yellow indicates tumor > background (hypermethylation), and blue indicates background > tumor (hypomethylation). The orange bar under the heatmap suggests a subgroup that shows relatively stronger hypermethylation, and the blue bar suggests a subgroup with remarkable hypomethlyation and low frequency of hypermethylated genes. **c**. Upper: a schema of the structure of a CpG island, and the definition of regions around such CpG islands. Middle: a schema of the structure of protein-coding genes, and relative positions of the terms related to distance from transcription start site. Lower: a schema of the structure of miRNA-coding regions. We defined “upstream” and “gene body” regions as shown in the figure. **d**. Relative methylation change (background levels subtracted from tumor levels) stratified by probe annotation (relative location from gene and CpG island: upstream/gene body/non-coding regions and CpG island/shore/shelf/non-island). **e**. Absolute methylation levels stratified by probe annotation (relative location from gene and CpG island). The gap between green and red lines indicates the difference in the methylation levels between background and tumor tissue. The gap was clearly observed in shelf/non-island regions and ranged from 10 to 20%

Tissues from a total of 43 patients were used for this study, including 13 (30.2%) females and 30 (69.8%) males. The mean age of patients was 68.1 years (standard deviation: 12.4). Non-supervised hierarchical clustering (1% randomly extracted) based on genome-wide DNA methylation distinguished NBNC-HCC samples clearly from their background non-tumor tissues (Fig. 1a). Tumor samples were characterized by hypomethylated CpGs (defined as a negative change of methylation level compared to respective background). Hierarchical clustering for the difference in methylation level between paired tumor and background tissues also showed commonly hypomethylated probes in cancer tissues (Fig. 1b). This suggests there are certain subgroups characterized by different methylation patterns (see orange and blue bars in Fig. 1b).

The functional impact of DNA methylation in respect to effects on gene expression differs depending on the characteristics of the respective CpG locus, such as distance from the transcription start site (TSS) and CpG island. We therefore stratified the data according to these factors based on the annotations defined by the Infinium HumanMethylation450 BeadChip Kit (summarized in Fig. 1c), and plotted the methylation change (tumor – background) in Fig. 1d. Methylation levels were highly downregulated for most sites except CpG islands within upstream regions (where generally promoters exist), where hypermethylation was dominantly observed (see the leftmost bar in Fig. 1d). Medians of the methylation change were distributed from –15.1% (non-island in non-coding region) to 0% (CpG island in any region). Absolute methylation levels in non-tumor background tissues were generally very high in gene body/3′UTR regions, and shelf/non-island regions. Indeed, the methylation levels in background tissues ranged from 70 to 90%, with tumor tissues exhibiting decreases of, on average, 10 to 20% of this level (Fig. 1e); on the other hand, changes of methylation levels were very discrete within CpG islands.

### Extensive DNA methylation alterations within miRNA coding and clustered miRNA regions

**Fig. 2:**
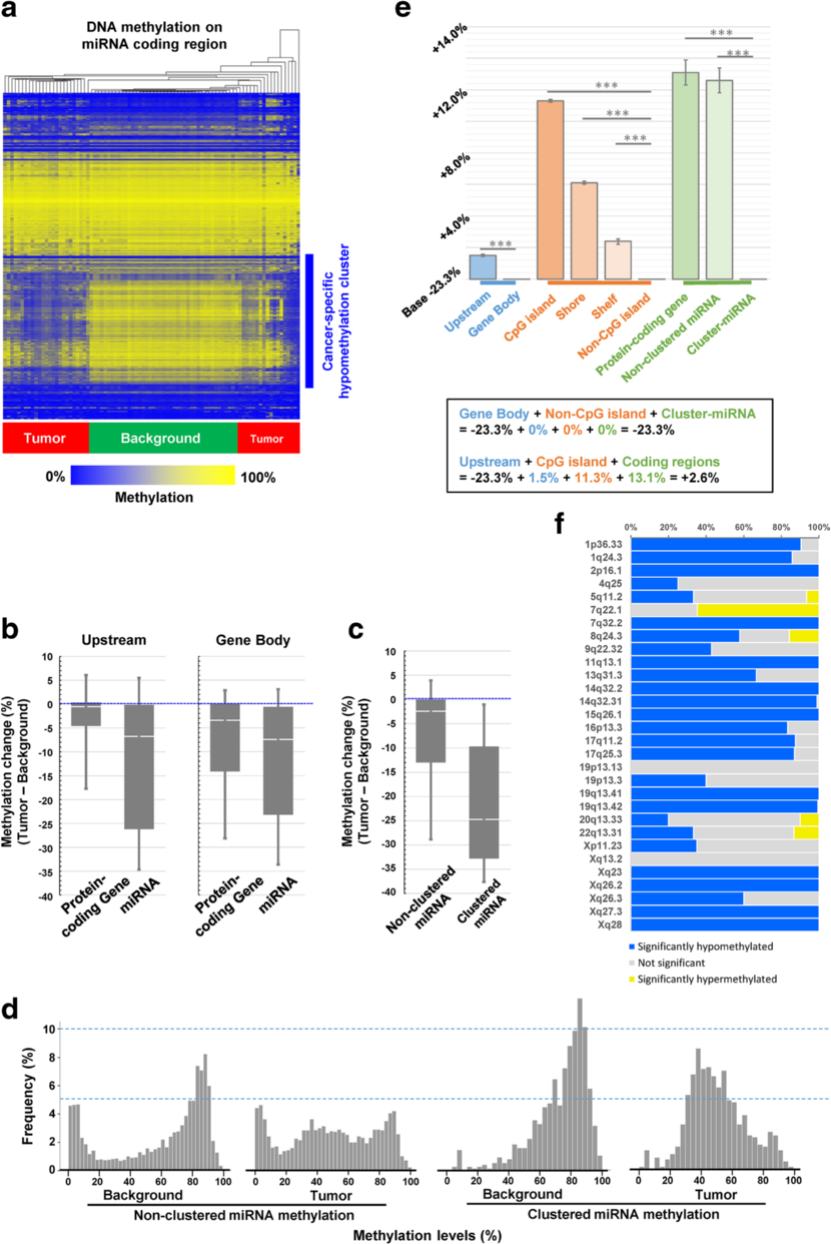
DNA methylation analyses for microRNA coding regions of tumor and background tissues in non-B non-C hepatocellular carcinoma patients. **a**. Hierarchical clustering of methylation data from tumor and background tissues relating to microRNA-coding regions. Methylation analysis was performed using the Infinium HumanMethylation450 BeadChip. All probes annotated within microRNA regions were used for the analysis. **b**. Relative methylation change (background levels subtracted from tumor levels) stratified by probe annotations (upstream regions/gene body and protein-coding gene/microRNA). **c**. Relative methylation change (background levels subtracted from tumor levels) stratified by probe annotations (non-clustered microRNAs/clustered microRNAs). **d**. Comparison of histogram plots showing methylation levels between background and tumor tissues stratified by non-clustered and clustered microRNA coding regions. **e**. Visualization of a statistical model showing methylation level in accordance to particular probe annotations (relative location from gene and CpG island, and protein-/miRNA-/clustered miRNA-coding). **f.** A proportion of significantly hypo/hyper-methylated CpGs within each miRNA cluster

As described above, hypomethylation was a common observation in tumor tissues. In particular, we detected marked hypomethylation within miRNA coding regions (Fig. 2). As shown in Fig. 2a, clustering on the basis of methylation of miRNA coding regions alone distinguished NBNC-HCC from background tissues, with remarkable evidence of a hypomethylation cluster being found. Methylation levels within miRNA coding regions were highly down-regulated compared to protein-coding gene regions, even when respective upstream regions were compared (Fig. 2b). Moreover, clusters of miRNAs, defined in this study as regions where three or more miRNAs were coded within 15,000 base pairs, were extremely hypomethylated in tumor samples (median for non-cluster miRNAs: -2.3%, cluster-miRNAs: -24.6%; Fig. 2c). To investigate this further, the distribution of methylation levels between tumor and background tissues, as well as between non-clustered and clustered miRNAs, were compared (Fig. 2d). As shown in Fig. 2d, the peak methylation level within the clustered miRNAs was clearly shifted from around 84% to 39%. The sharpness of the peaks and the peak shift was more obvious within clustered miRNAs.

In fact, 66.2% of the CpG probes within cluster miRNAs were annotated to non-island areas, a highly hypomethylated region, in the tumor. As the proportions of non-island areas were only 36.3% and 49.7% within all probes and all miRNAs, respectively, this imbalance can cause a bias. However, using statistical modeling approaches, it was suggested that the methylation level of the clustered miRNAs was significantly lower than that observed with protein-coding genes and non-clustered miRNAs, which was independent of the distance from the TSS and the CpG island (Additional file 1: Table S1, Fig. 2e). In Fig. 2f, we investigated a proportion of significantly hypomethylated CpGs within each miRNA cluster. As a result, 80% of CpGs were significantly hypomethylated (paired-t test, *P* < 0.05) within 56.7% (17/30) of miRNA clusters (17/30, 56.7%).

### Regulation of global miRNA expression by DNA methylation within miRNA coding regions

**Fig. 3:**
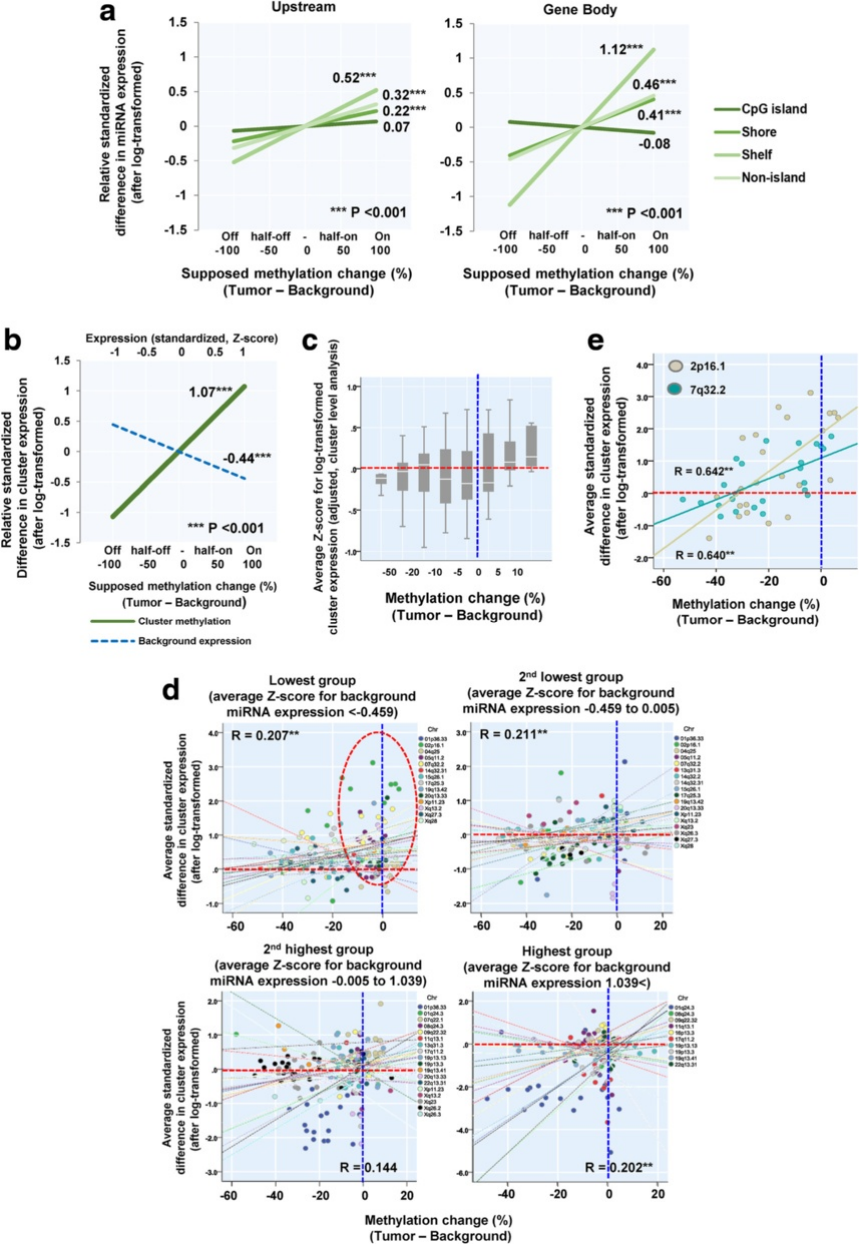
Association between methylation levels of microRNA-coding regions and respective microRNA expression. **a**. Visualization of a statistical model relating microRNA expression with respective methylation change, stratified by relative location from gene and CpG island. The effect of methylation change was estimated from regression coefficients for the relative methylation change. Methylation and expression levels in background tissues were also included as a covariate. Darkest line: CpG island, darker: Shore, lighter: Shelf, lightest: Non-island. **b**. Visualization of a statistical model relating average clustered microRNA expression with respective methylation change (cluster-level analysis). The effect of methylation change was estimated from regression coefficients for the relative methylation change. Methylation and expression levels in background tissues were also included as a covariate. Dark green line: Clustered miRNA methylation, blue dotted: background expression of clustered miRNAs. **c**. Box plots for correlation between clustered microRNA expression and methylation change. **d**. Scatter plots for correlation between average expression and methylation of microRNA cluster 2p16.1 and 7q32.2. Gray: 2p16.1, green: 7q32.2. **e**. Relative methylation change (backgroun. Scatter plots and associated regression regarding correlation between clustered microRNA expression and methylation stratified by quartile of microRNA expression in background tissues. Circles were painted in different colors according to miRNA clusters

We subsequently assessed the association between DNA methylation within miRNA coding regions and expression of respective miRNAs, with baseline level of expression and methylation also taken into consideration. As shown in Additional file 1: Table S2 and Fig. 3a, we constructed and utilized a statistical model (linear mixed model) for this assessment. The model indicated that DNA methylation changes were generally positively associated with corresponding differences in expression of the respective miRNAs. In particular, following statistical adjustment of miRNA methylation and expression in corresponding background tissues, altered methylation levels of CpG sites in shore, shelf and non-island areas within miRNA coding regions were significantly associated with expression changes of the relevant miRNAs. The expression of conventional protein-coding genes was negatively associated with methylation of CpG islands within upstream regions; however, this association was not observed in the case of miRNA expression.

### Varied, albeit globally positive correlation, between expression and methylation within clustered miRNAs

Almost half of miRNAs are clustered together, which demonstrated impressive hypomethylation in tumor tissues (Fig. 2d). Methylation and expression levels were similarly controlled amongst closely-located miRNAs, with the number of CpG sites involved in transcriptional regulation being relatively small, compared to protein-coding genes (Additional file 2: Figure S1). This suggests that transcriptional regulation via methylation in miRNA coding regions is possibly more visible with cluster-level summarized data. Thus, we then focused on analysis of cluster-level regulation of expression. Summary data regarding methylation and expression levels for miRNA clusters is shown in Additional file 1: Table S3.

As shown in Additional file 3: Figure S2A, methylation and expression levels of the same miRNA cluster were quite similar across all of the background tissues. However, across the tumor tissues, this similarity was disturbed by demethylation tendency within miRNA clusters, with methylation levels in tumor tissues being relatively more varied in nature. A statistical model providing cluster level analysis (constructed in a similar way to that shown in Fig. 3a) also suggested a positive correlation between average change in methylation and expression within each cluster (Additional file 1: Table S4, Fig. 3b). Figure 3c shows in detail the distribution of average miRNA cluster expression according to different methylation levels. Figure 3d also shows the association between differences in methylation and expression of miRNA clusters, when taking into consideration confounding effects due to background tissue expression. In every stratum stratified by the quartile of background tissue expression, the majority of clusters showed a positive association between methylation and expression change of clustered miRNAs. Pearson’s correlation coefficients of their association were *R* = 0.144 to 0.211 (not strong, but significant with *P* < 0.05 in 3/4 strata). The red circle in the figure indicates up-regulated clusters found within the lowest background expression group, with remarkable up-regulation observed in samples displaying 0% of difference in methylation (i.e. no methylation change). Even though tumor-specific hypomethylation and up-regulation of miRNA cluster expression were observed within the same miRNA cluster, expression was seemingly paradoxically up-regulated in certain cases within the intact-methylation group. For example, miRNA clusters on 2p16.1 and 7q32.2 were, respectively, the first and 2^nd^ most up-regulated miRNA clusters across all the samples; both clusters were highly hypomethylated, but tumor-specific upregulation was observed in samples with no methylation change (Fig. 3e).

**Fig. 4:**
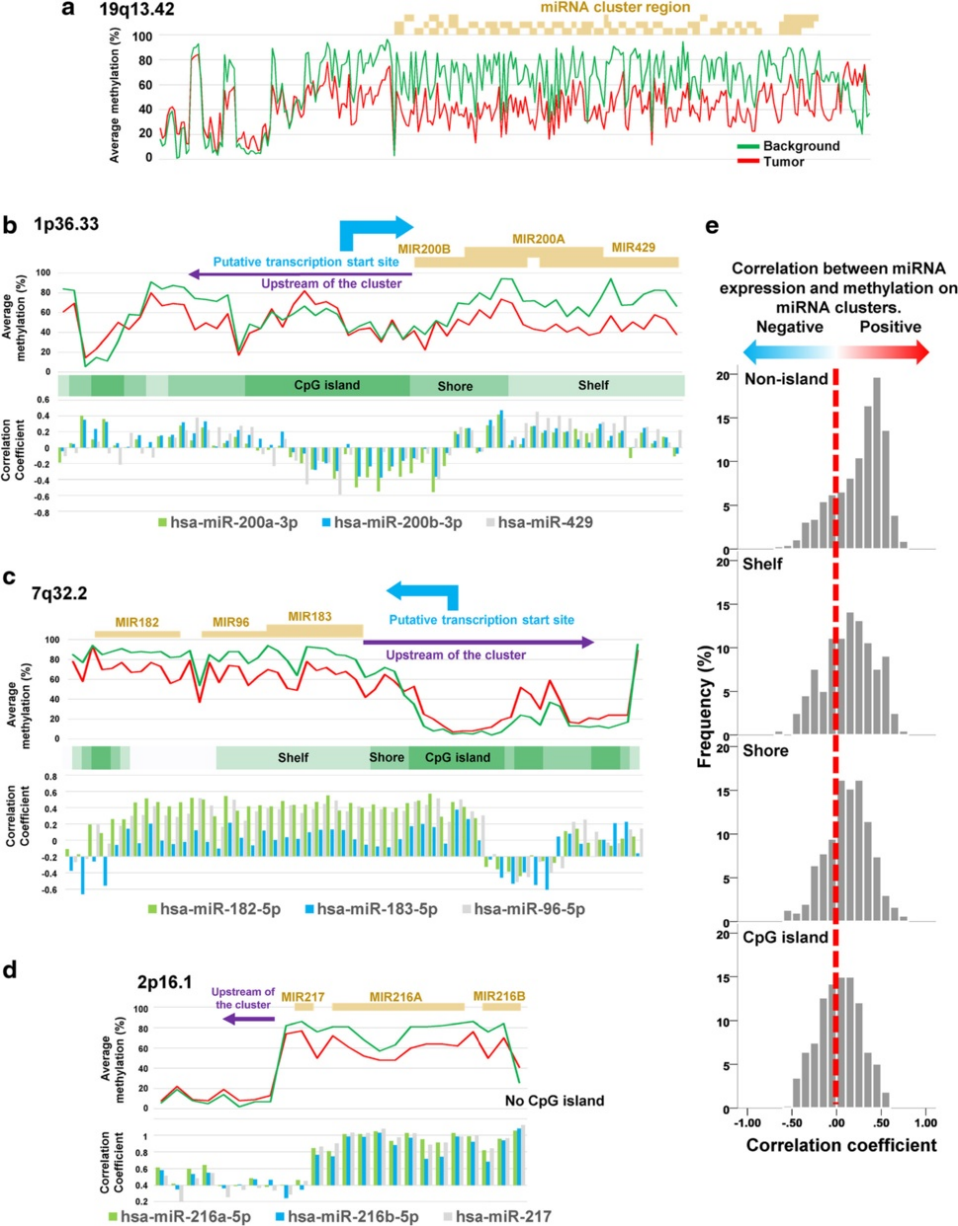
Methylation level for each probe and transcriptional regulation of microRNA clusters. **a**. Average methylation levels of each probe around the largest microRNA cluster on 19q13.42. Dark yellow bars indicate the miRNA coding regions. **b**-**d**. Average methylation levels and correlation coefficients between methylation level and microRNA expression around microRNA cluster on 1p36.33 (B), 7q32.2 (C), and 2p16.1 (D). **e**. Histogram plots of correlation coefficients between average expression of microRNA clusters and methylation level of miRNA clusters including upstream from clusters within 20k bps stratified by relative location from CpG island

The detailed methylation changes around the largest cluster on 19q13.42 is shown in Fig. 4a. A remarkable hypomethylation pattern was specifically observed within this miRNA cluster, with methylation differences fading when out of the cluster region. Subsequently, we assessed the extent of correlation between methylation and expression more precisely (at a single probe scale) within several miRNA clusters. Since regulation of the miRNA clusters on 1p36.33 (the 2^nd^ most down-regulated) and 7q32.2 (the 2^nd^ most up-regulated) have been reported in detail [20, 21], we selected these for focused analysis. As shown in Fig. 4b, the methylation levels within miRNA cluster on 1p36.33 was positively correlated with expression of its linked miRNAs. However, this miRNA cluster also has a large CpG island in the upstream region of the cluster within 20k bps, with methylation level in this area negatively correlated with expression of miRNAs within the cluster. A similar regulatory pattern was also observed in the case of the miRNA cluster on 7q32.2 (Fig. 4c). The miRNA cluster on 2p16.1, where there is no CpG island within the upstream region of the cluster, demonstrates only a simplistic positive correlation (Fig. 4d). Correlation coefficients determined between methylation and expression for miRNA clusters with upstream sequences within 20k bps are summarized in Fig. 4e. Consistent with the analysis shown for 2p16.1, methylation at CpG sites in non-island areas was highly positively correlated with expression. CpG islands were mainly located within upstream regions of miRNA clusters (68.8%), with approximately half of these showing a negative correlation in terms of methylation status with expression, such as that evidenced in Fig. 4b and c. Methylation-expression correlation analysis at a chromosome-wide scale is summarized in Additional file 3: Figure S2B, showing correlation coefficients are varied in nature (positive to negative) depending on location.

### Ability for tumor discrimination: comparison of expression and methylation data

**Fig. 5:**
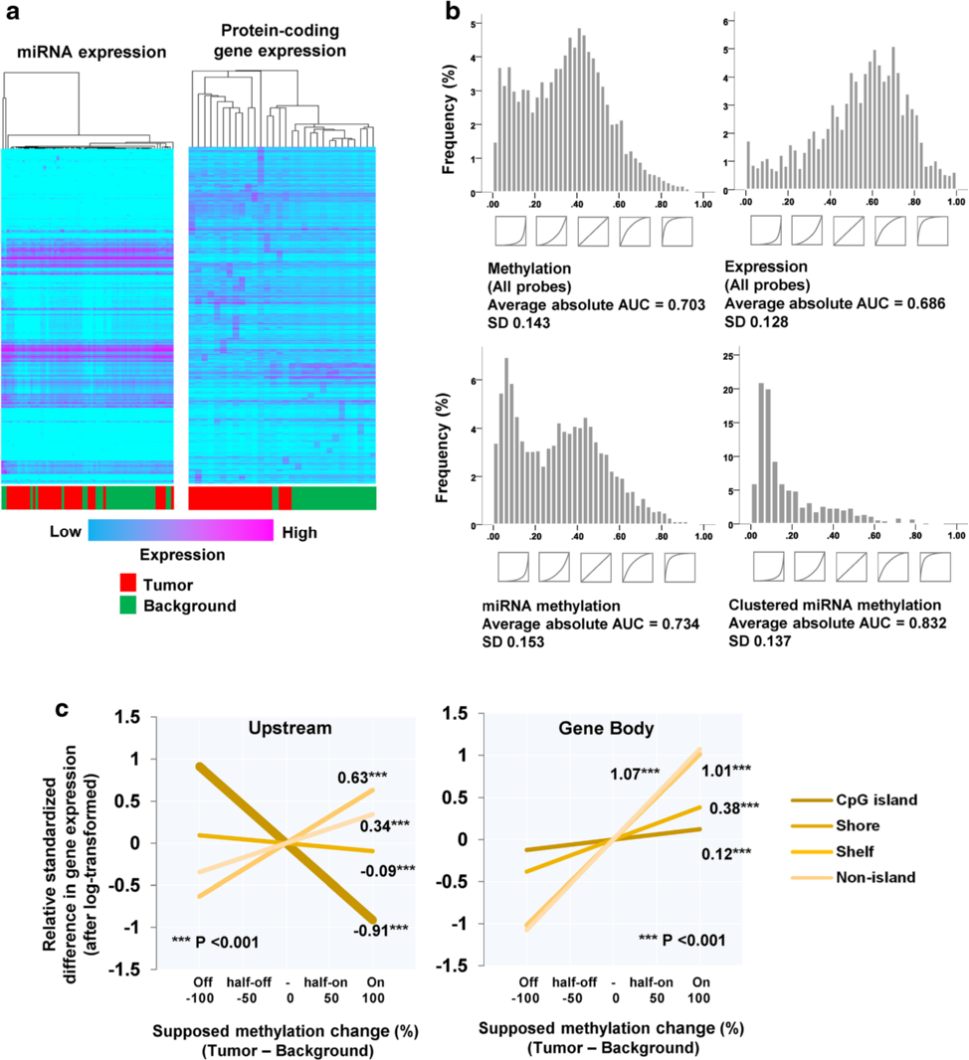
Assessment of ability for tumor discrimination, and regulation of protein-coding gene expression by methylation. **a**. Hierarchical clustering of genome-wide microRNA and protein-coding gene expression analysis data from tumor and background tissues. One percent of probes were randomly sampled for the analysis of protein-coding genes. **b**. Histogram plots of area under curves (AUC) of repeated receiver operator characteristic (ROC) analysis in respect of ability to discriminate tumor from background tissue. Curves shown below are images of representative results from ROC analysis for various probes. **c**. Visualization of a statistical model relating expression of protein-coding genes with respective methylation change stratified by relative location from gene and CpG island. The effect of methylation change was estimated from regression coefficients for the relative methylation change. Methylation and expression levels in background were also included as covariate. Darkest line: CpG island, darker: Shore, lighter: Shelf, lightest: Non-island

A major perceived impact in respect to methylation-dependent influences on tumorigenesis is thought to relate to regulation of gene expression. However, clustering using genome-wide expression data, as shown in Fig. 5a, provided little evidence of tumor versus background tissue discriminatory power, in contrast to the potential power of methylation data to produce a robust separation (as seen in Fig. 1a). Ability to provide tumor versus background discrimination was quantified using ROC analysis for every protein-coding gene and miRNA probe (in terms of both methylation and expression). Accordingly, a great discriminatory ability was shown when data from clustered miRNA methylation was utilized (Fig. 5b). Consistent with the results shown in Fig. 5a, methylation was superior to expression in respect to discriminatory power.

### Significance of DNA methylation alteration within miRNA coding regions for target gene expression: a genome-wide integrative analysis

For comparison purposes, a statistical model for protein-coding genes was then constructed and analysed (Additional file 1: Table S5, Fig. 5c). As previously known, methylation differences in CpG islands within upstream regions was significantly negatively correlated with relative gene expression (regression coefficient = -0.91: decreasing -0.91 x SD when 100% methylation increase, *P* < 0.001), with this correlation pattern gradually changing to positive when progressing further from the CpG island. On the contrary, methylation within gene body regions positively regulated expression, particularly in CpG-sparse regions (shelf and non-island CpGs). Additionally, we assessed the association between expression change of several DNMTs (DNMT1, DNMT3A, DNMT3B) and average methylation difference of clustered miRNAs. Correlation coefficients for z-transformed log-ratio of DNMTs and methylation difference were 0.135 for DNMT1, 0.375 for DNMT3A, 0.093 for DNMT3B.

**Fig. 6:**
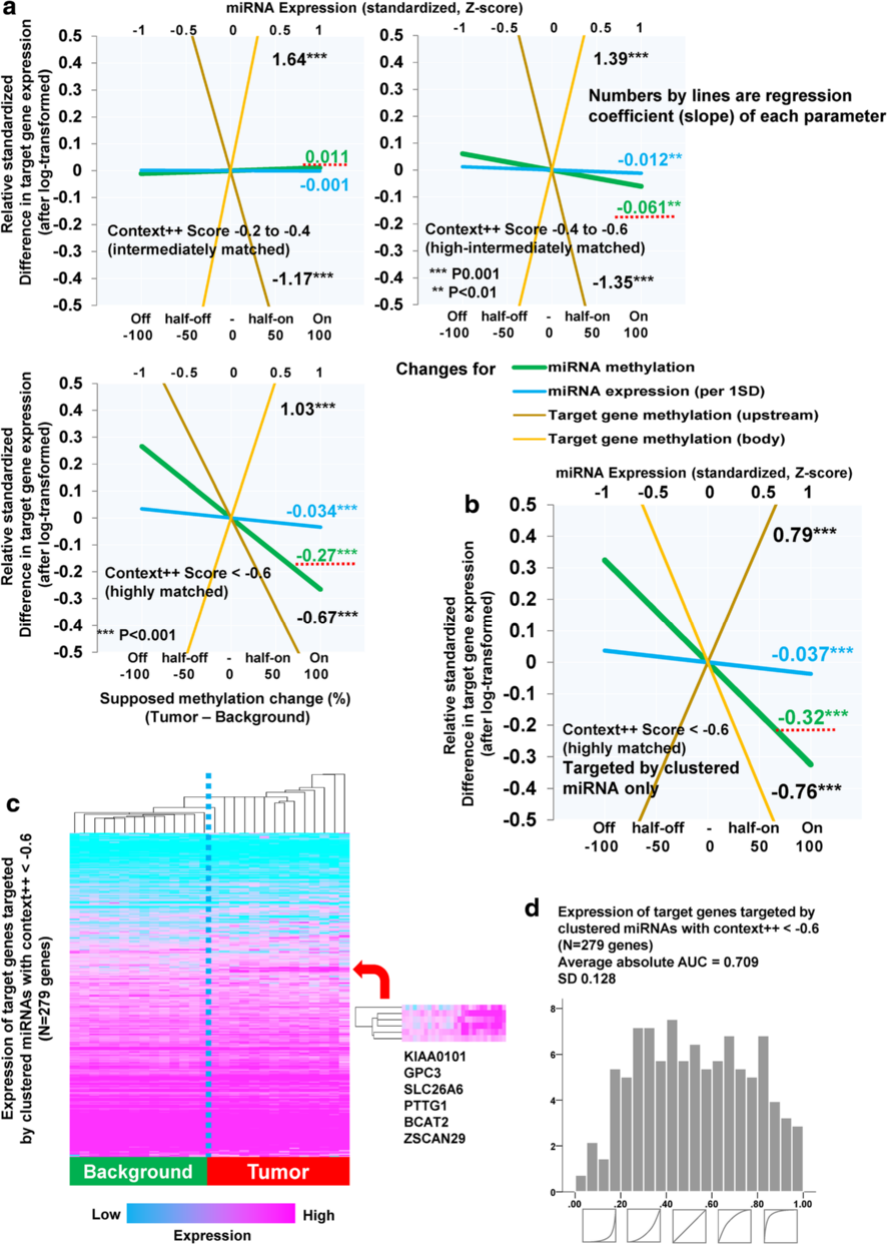
Regulation of target gene expression depending on miRNA methylation. **a**. Visualization of a statistical model relating target gene expression with methylation change of corresponding microRNAs stratified by three different levels of context++ score, which is an index determining the degree of matching. The effect of methylation change was estimated from coefficients for the relative methylation change. Methylation and expression levels of microRNAs in background tissues, and target gene methylation were also included as covariates. Green line: miRNA methylation, blue: miRNA expression, dark yellow: target gene methylation (promoter), yellow: target gene methylation (body). **b**. A similar analysis to Fig. 6a only applying the target genes targeted by clustered miRNAs within highly matched miRNA sites (context++ score < -0.6). **c**. Hierarchical clustering of expression of target genes targeted by clustered miRNAs with context++ score < -0.6. Blue dotted line is a border for tumor-background. A red arrow indicates a cluster characterized by tumor-specific overexpression of target genes. **d**. A similar analysis to Fig. 5b only applying target genes targeted by clustered miRNAs with context++ score < -0.6 (highest matching)

Finally, we assessed the significance of dynamic hypomethylation in miRNA coding regions for target gene expression in the context of tumor development. As described above, the ability to provide tumor versus background tissue discrimination was easier to achieve when utilizing the methylation status of miRNA coding regions than expression levels of particular miRNAs. We, therefore, suggest that methylation status is also a better estimator of target gene expression than expression of the respective miRNA. Target genes for each miRNA was determined based on TargetScan 7.0 (www.targetscan.org). For this investigation, we selected all conserved miRNA sites and corresponding target genes with greater context++ score (index for degree of matching: lower than -0.2) [22]. We then used a modeling approach similar to that shown in Fig. 5c, but including changes in miRNA methylation, miRNA expression, and target gene methylation (upstream region and gene body) and their absolute values in background tissues. Also, taking into account the affinity of miRNA sites are dependent on the context++ score, we stratified the analysis according to three different levels of context++ score (results summarized in Fig. 6a and Additional file 1: Table S6). Amongst the highly matched miRNA sites (context++ score < -0.6), miRNA methylation changes were significantly negatively correlated with corresponding target gene expression change independently from miRNA expression (regression coefficient: -0.27, *P* < 0.001; decreasing -0.27 x SD in the case of 100% methylation increase). Absolute value of regression coefficients gradually increased in relation to the context++ score (degree of matching). These data suggest that miRNA hypomethylation is associated with up-regulation of target gene expression.

In addition, to identify the influence of the clustered miRNAs, we performed a similar analysis to Fig. 6a only applying the target genes that were targeted by the clustered miRNAs within highly matched miRNA sites (context++ score < -0.6). As shown in Fig. 6b (and Additional file 1: Table S6), we observed the highest regression coefficient for miRNA methylation change than any previous analyses (Regression coefficient: -0.32, *P* < 0.001). This suggests that the influence of methylation change is more efficient among target genes that are targeted by high-affinity clustered miRNAs. Subsequent hierarchical clustering using such highly-sensitive target genes demonstrated much better separation of tumor from background tissues than in Fig. 5a, showing tumor-specific overexpression of several target genes in which an established oncogene, GPC3, for hepatocellular carcinoma was included (Fig. 6c). A similar analysis to Fig. 5b for the same target genes also showed better ability for tumor discrimination (Fig. 6d), compared to the result of non-specified gene expression (see the right upper graph in Fig. 5b). These results suggest an importance of miRNA methylation change for tumor development or progression via regulation of their target gene expression.

## Discussion

Our integrated analyses of methylome and transcriptomic data revealed highly tumor-specific hypomethylation of clustered miRNAs in NBNC-HCCs. The methylation pattern of clustered miRNAs between background and tumor tissues were much more discriminative than expression data from protein-coding genes or miRNAs. The expression change of miRNAs was, on average terms, positively associated with methylation change in the corresponding coding region of miRNA; however, this pattern of regulation was not simple and depended on the relative location from genes. Finally, our result indicated an independent association in respect of miRNA methylation alteration with target gene expression changes from background to tumor tissue. The findings suggest that the broad tumor-specific hypomethylation in miRNA coding regions functionally influence tumor development through regulating target gene expression.

Global hypomethylation is established epigenetic alteration in tumor cells [14, 15]. Cluster miRNA hypomethylation seems to be happening independently from non-specific global hypomethylation (Fig. 2e). Clustered miRNA hypomethylation has been reported by several studies; however, its significance and relevance to regulation of gene expression is not well understood [23–26]. Jeong M et.al. reported DNMT3A as being key to clarify and ensure distinctions between low and high DNA methylation areas in the genome [27]. However, in our data, even though DNMT3A expression was highly upregulated, total methylation levels were down-regulated in the tumor samples although a slight positive correlation was observed between global methylation level and expression of each DNMT. The gene encoding the TET protein, which was recently identified to be involved in DNA demethylation [28], was not found as a key factor for tumor-specific hypomethylation in this study. Nevertheless, we could not identify the cause of global hypomethylation observed within NBNC-HCC tissues in this study.

Several studies have reported that hypomethylation of miRNA clusters is associated with re-activation of corresponding miRNA expression [24, 25, 29]. However, we could not conclude that tumor-specific hypomethylation was simply associated with tumor-specific expression (see Additional file 3: Figure S2A and Fig. 3c, d). Paradoxically, up-regulation mostly occurred in methylation-intact samples and the expression of hypomethylated samples was not overly up-regulated. It can be explained via the influence of other factors involved in transcription regulation, such as transcription factors, chromosome amplification/deletion, etc. Even if transcriptional signals were turned into “on” through tumor development, if methylation of miRNA coding regions positively regulated affinity downstream of the signal, results as seen for Fig. 3d may be observed.

In addition, as shown in Fig. 4b and c, expression of miRNA clusters on 1p36.33 and 7q32.2 was negatively correlated with CpG island methylation within the upstream regions of the clusters, and positively correlated with methylation in miRNA-coding regions. This finding is consistent with previous reports [20, 21]. It indicates that a typical regulation pattern for protein-coding genes was found within both clusters, which is characterized by transcriptional regulation depending on methylation in promoter CpG islands. It also suggests that the difference in the transcriptional correlation of methylation change within upstream regions between each miRNA-coding region (please note it is different from “upstream region of the cluster”) and protein-coding genes (defined as Fig. 1c, and compare Figs. 3a, 4 and 5c) could be due to the difference in promoter activity of such regions. However, the correlations observed in the upstream regions of the clusters (see Fig. 4b, and c) are not necessarily representative pattern of miRNA transcription regulation (see the distribution of correlation coefficients in Fig. 4e). A similar inconsistent phenomenon was observed in the CTCF binding site. The CTCF binding site is considered important for miRNA expression regulation, as well as DNA methylation, and it modifies correlation patterns positively and negatively between expression and methylation [18, 30].

Although aberrant methylation within promoter-bound CpG islands has been well studied for some time, the significance of gene body methylation and non-CpG island areas has until recently been minimized. Our results suggested the significance is not that small compared to promoter CpG islands, being consistent with recent reports about positive correlation between gene body methylation and expression [19, 31]. In tumor samples, large portions of gene bodies were relatively highly hypomethylated, and the relationship between this and tumor development should be studied. more Yang X et. al. described that gene body methylation may lead to genome instability and reduced efficiencies of post-transcriptional activity; other reports also support this idea [15]. Similar to miRNA regulation, direction of correlation between methylation of gene body and expression varies across the genome [13, 31].

In Fig. 6, miRNA methylation seems to regulate target expression directly amongst highly matched miRNA sites as it were independent from miRNA expression. Such results may be due to instability of expression measurement especially for miRNA, and stability of methylation measurement. In addition, transcription is regulated by multiple factors and dynamically changes moment by moment, with miRNAs also affecting surrounding/other cells by forming exosomes [32]. These findings might provide some explanation towards the discriminatory capability of tumor discrimination being present when methylation is considered (Fig. 5b). Moreover, our results in Fig. 6 indicate that expression of target genes of highly-matched clustered miRNAs differs between tumor and background tissues. This could be attributable to drastic hypomethylation of miRNA clusters. In general, global hypomethylation develops gradually through tumor development. For example, the pre-myeloma condition MGUS (Monoclonal gammopathy of undetermined significance) demonstrated intermediate methylation levels between normal and myeloma cells [15]. Pre-cancerous infections (e.g. HBV, HCV) also affect global methylation level in a similar way [10, 12]. In addition, an established oncogene of hepatocellular carcinoma, GPC3 [33], was included in these target genes (Fig. 6c), and showed a tumor-specific overexpression pattern. Taken together, we consider that broad hypomethylation of miRNA clusters develops gradually through tumorigenesis, and not only as a specific phenotype but also a functionally significant factor.

## Conclusion

We observed a dramatic negative shift in methylation levels within miRNA cluster regions, and investigated ways to correlate with expression data. Methylation changes in miRNAs were more indicative for target gene expression than miRNA expression change itself, suggesting the importance of genome-wide miRNA methylation for cancer development. Our study dynamically summarized the global miRNA hypomethylation and its genome-wide consequence in NBNC-HCC.

## Methods

### Subjects and sample collection

All subjects (*N* = 43) were diagnosed as HCC without HBV or HCV infection by expert hepatologists from Jan. 2004 to Dec. 2013. After informed consent for research was given, histologically diagnosed paired tissue samples (HCC and background liver) were collected from surgery samples at Osaka City University Hospital, Kyushu University Hospital, Tokushima University Hospital, and Tohoku University Hospital. This study was firstly approved by Nagoya City University ethical board as the primary center of the study (the submission number: 805-2), followed by ethical boards in each of the participating hospitals.

### Comprehensive analyses for DNA methylation and miRNA/mRNA expression

The DNeasy Blood & Tissue Kit (QIAGEN, USA) was used for DNA extraction, and mirVana miRNA Isolation Kit (Ambion, USA) and miRNeasy Mini Kit (QIAGEN, USA) was used for RNA extraction (the former was used for miRNA extraction, the latter was for total mRNA extraction). All collected samples were large enough to avoid major contamination of background tissue into tumor samples (median major axis: 47mm, interquartile range: 29-67.5mm, range: 16 to 170mm). Genome-wide DNA methylation status was assessed by an Infinium HumanMethylation450 BeadChip Kit (Illumina, USA, *N* = 43 pairs of tumor and background). Genome-wide mRNA expression analysis was performed using a SurePrint G3 Human GE 8x60K c2 Microarray (Agilent, USA, *N* = 15 pairs of tumor and background). These analyses were performed at the CDM center, Takara Bio Inc. (Shiga, Japan). A 3D-Gene® Human miRNA Oligo Chip V20 (Toray, Japan, *N* = 24 pairs of tumor and background) was used for comprehensive miRNA expression analysis. Data files for these genome-wide experiments were submitted to ArrayExpress (https://www.ebi.ac.uk/arrayexpress/) under accession numbers of E-MTAB-4169, E-MTAB-4171, and E-MTAB-4170, respectively. All analyses were performed according to the procedure provided by the manufacturer. Target genes for miRNAs were identified using TargetScan 7.0 (http://www.targetscan.org). We used all conserved miRNAs for human transcripts with greater context++ score (less than -0.2) [22].

### Statistical analyses

General data mining and statistical analyses were performed via use of SPSS 20 (IBM, USA). Genome-wide methylation analysis was performed using beta values for all CpG loci represented on the Infinium HumanMethylation450BeadChip. For integrated analyses, the average methylation level or each gene (also stratified by upstream region/gene body and CpG island/shore/shelf/non-island) was calculated, and then average expression of each gene (log-transformed signal and log-ratio) and average expression of each miRNA were annotated according to the corresponding methylation data. Clustering was performed using Cluster 3 [34]. Values measured by comprehensive analyses were transformed logarithmically and z-standardized as necessary. Continuous variables were generally compared by t-test or paired t-test. Area under curve (AUC) was calculated by receiver operating characteristic (ROC) analysis. General linear and liner mixed models were used for statistical modeling for determining expression-methylation association. In the mixed models, a patient identifier was included as a random effect. The other variables indicated in figures and tables were included as fixed effects. *P* < 0.05 was considered significant.

### Availability of supporting data

The data sets supporting the results of this article are available in the ArrayExpress repository, under accession numbers of E-MTAB-4169, E-MTAB-4170, and E-MTAB-4171 in https://www.ebi.ac.uk/arrayexpress/.

## Additional files

Additional file 1:
**Table S1.** A general linear model for average methylation change attributed to genomic annotations of the probes. **Table S2.** A linear mixed model for miRNA expression change between tumor and background tissues attributed to their methylation change stratified by distance from CpG island and transcription start site. **Table S3.** Summary for methylation and expression of miRNA clusters. (Expression difference was z-transformed after obtaining log-ratio between background and tumor tissues. Thus, it was not equal to simple subtraction of background from tumor). **Table S4.** A linear mixed model for miRNA cluster expression (average within each cluster) change attributed to average cluster methylation change. **Table S5.** A linear mixed model for expression change of general protein-coding genes between tumor and background tissues attributed to their methylation change stratified by distance from CpG island and transcription start site. **Table S6.** A linear mixed model for target gene expression change between tumor and background tissues attributed to corresponding miRNA methylation change. (DOC 252 kb) Additional file 2: Figure S1. Correlation coefficients between methylation/expression change of microRNAs within selected microRNA clusters (1p36.31, 1q24.3, 2p16.1, and 7q32.2). The selected clusters consist of the most and 2^nd^ most up-/down- regulated clusters (see Supplementary Table S3). (TIFF 3351 kb) Additional file 3: Figure S2. Association between methylation levels of microRNA-coding regions and microRNA expression. A. Scatter plots showing correlation between clustered microRNA expression and methylation stratified by background and tumor tissues. B. Chromosome-wide correlation coefficients between methylation level as determined by each probe and corresponding microRNA expression. (TIFF 2122 kb)

## Abbreviations

AUC: Area under curve
HCC: Hepatocellular carcinoma
HBV: Hepatitis B virus
HCV: Hepatitis C virus
NAFLD: Non-alcoholic fatty liver disease
NBNC-HCC: Non B non C hepatocellular carcinoma
miRNA: microRNA
mRNA: messenger RNA
SD: Standard deviation
ROC: Receiver operating characteristics
