# Supplementary material for: Global, cancer-specific microRNA cluster hypomethylation was functionally associated with the development of non-B non-C hepatocellular carcinoma
Source: Mol Cancer. 2016 Apr 30;15:31. doi: 10.1186/s12943-016-0514-6 (PMC4852433; doi:10.1186/s12943-016-0514-6)
Supplement: Additional file 1: — Table S1. A general linear model for average methylation change attributed to genomic annotations of the probes. Table S2. A linear mixed model for miRNA expression change between tumor and background tissues attributed to their methylation change stratified by distance from CpG island and transcription start site. Table S3. Summary for methylation and expression of miRNA clusters. (Expression difference was z-transformed after obtaining log-ratio between background and tumor tissues. Thus, it was not equal to simple subtraction of background from tumor). Table S4. A linear mixed model for miRNA cluster expression (average within each cluster) change attributed to average cluster methylation change. Table S5. A linear mixed model for expression change of general protein-coding genes between tumor and background tissues attributed to their methylation change stratified by distance from CpG island and transcription start site. Table S6. A linear mixed model for target gene expression change between tumor and background tissues attributed to corresponding miRNA methylation change. (DOC 252 kb) [file 12943_2016_514_MOESM1_ESM.doc]

Supplementary Table S1.

A general linear model for average methylation change attributed to genomic annotations of the probes.

|  |  |  |  | 95%CI | |
| --- | --- | --- | --- | --- | --- |
|  | Parameter | Estimate | P value | Lower | Upper |
|  | Intercept | -0.233 |  | -0.241 | -0.225 |
| Location | Gene Body | -0.015 | <0.001 | -0.015 | -0.014 |
|  | Promoter | Reference |  |  |  |
| CpG status | CpG island | 0.113 | <0.001 | 0.113 | 0.114 |
|  | Shore | 0.061 | <0.001 | 0.060 | 0.062 |
|  | Shelf | 0.024 | <0.001 | 0.023 | 0.026 |
|  | non-CpG island | Reference |  |  |  |
| Gene | Gene-coding region | 0.131 | <0.001 | 0.123 | 0.139 |
|  | miRNA cluster | 0.126 | <0.001 | 0.118 | 0.135 |
|  | miRNA-coding region | Reference |  |  |  |
| Dependent variable: average methylation change (background - tumor) | | | | |  |

Supplementary Table S2.

A linear mixed model for miRNA expression change between tumor and background attributed to their methylation change stratified by distance from CpG island and transcription start site.

|  |  |  |  |  | 95% confidence interval | |
| --- | --- | --- | --- | --- | --- | --- |
| CpG status | Location | Parameter | Estimate | P value | Lower | Upper |
| CpG island | Gene Body | Intercept | 0.113 |  | 0.056 | 0.170 |
|  |  | miRNA methylation change | 0.068 | 0.067 | -0.005 | 0.140 |
|  |  | miRNA methylation in background | -0.070 | <0.001 | -0.106 | -0.033 |
|  |  | miRNA expression in background | -0.228 | <0.001 | -0.240 | -0.216 |
|  | Promoter | Intercept | 0.020 |  | -0.040 | 0.080 |
|  |  | miRNA methylation change | -0.079 | 0.297 | -0.226 | 0.069 |
|  |  | miRNA methylation in background | 0.066 | 0.055 | -0.001 | 0.134 |
|  |  | miRNA expression in background | -0.221 | <0.001 | -0.242 | -0.201 |
| Shore | Gene Body | Intercept | 0.121 |  | 0.073 | 0.168 |
|  |  | miRNA methylation change | 0.218 | <0.001 | 0.150 | 0.287 |
|  |  | miRNA methylation in background | -0.133 | <0.001 | -0.175 | -0.091 |
|  |  | miRNA expression in background | -0.237 | <0.001 | -0.249 | -0.226 |
|  | Promoter | Intercept | -0.094 |  | -0.173 | -0.015 |
|  |  | miRNA methylation change | 0.406 | <0.001 | 0.244 | 0.568 |
|  |  | miRNA methylation in background | 0.196 | <0.001 | 0.098 | 0.293 |
|  |  | miRNA expression in background | -0.442 | <0.001 | -0.475 | -0.409 |
| Shelf | Gene Body | Intercept | -0.078 |  | -0.158 | 0.002 |
|  |  | miRNA methylation change | 0.522 | <0.001 | 0.402 | 0.642 |
|  |  | miRNA methylation in background | 0.124 | 0.008 | 0.032 | 0.216 |
|  |  | miRNA expression in background | -0.272 | <0.001 | -0.290 | -0.253 |
|  | Promoter | Intercept | -0.097 |  | -0.245 | 0.051 |
|  |  | miRNA methylation change | 1.121 | <0.001 | 0.850 | 1.392 |
|  |  | miRNA methylation in background | 0.106 | 0.299 | -0.094 | 0.305 |
|  |  | miRNA expression in background | -0.254 | <0.001 | -0.292 | -0.216 |
| Non-CpG island | Gene Body | Intercept | 0.026 |  | -0.013 | 0.066 |
|  |  | miRNA methylation change | 0.316 | <0.001 | 0.279 | 0.354 |
|  |  | miRNA methylation in background | 0.007 | 0.749 | -0.037 | 0.051 |
|  |  | miRNA expression in background | -0.318 | <0.001 | -0.325 | -0.311 |
|  | Promoter | Intercept | 0.108 |  | 0.029 | 0.186 |
|  |  | miRNA methylation change | 0.460 | <0.001 | 0.379 | 0.542 |
|  |  | miRNA methylation in background | -0.066 | 0.151 | -0.156 | 0.024 |
|  |  | miRNA expression in background | -0.391 | <0.001 | -0.408 | -0.374 |
| Random effect: individual ID, and expression data were z, and log-transformed.  Dependent variable: miRNA expression change (background - tumor), z, and log-transformed | | | | |  |  |
|  |  |

Supplementary Table S3.

Summary for methylation and expression of miRNA clusters

|  | Methylation level | | | Expression level (z, log-transformed) | | |
| --- | --- | --- | --- | --- | --- | --- |
| Location of miRNA cluster | Background | Tumor | Difference | Background | Tumor | Difference* |
| 1p36.33 | 0.694 | 0.510 | -0.184 | 0.084 | -0.431 | -1.054 |
| 1q24.3 | 0.793 | 0.622 | -0.170 | 1.557 | 0.696 | -1.794 |
| 2p16.1 | 0.797 | 0.615 | -0.182 | -0.554 | -0.133 | 0.815 |
| 4q25 | 0.844 | 0.845 | 0.001 | -0.672 | -0.693 | -0.031 |
| 5q11.2 | 0.473 | 0.403 | -0.070 | -0.728 | -0.653 | 0.166 |
| 7q22.1 | 0.697 | 0.746 | 0.049 | 0.674 | 0.882 | 0.396 |
| 7q32.2 | 0.837 | 0.623 | -0.214 | -0.533 | -0.364 | 0.412 |
| 8q24.3 | 0.826 | 0.805 | -0.021 | 0.983 | 1.038 | 0.091 |
| 9q22.32 | 0.866 | 0.851 | -0.015 | 1.081 | 1.078 | -0.057 |
| 11q13.1 | 0.440 | 0.380 | -0.060 | 1.361 | 1.323 | -0.146 |
| 13q31.3 | 0.300 | 0.221 | -0.079 | 0.682 | 0.755 | 0.084 |
| 14q32.2 | 0.840 | 0.541 | -0.299 | -0.177 | -0.133 | 0.089 |
| 14q32.31 | 0.783 | 0.460 | -0.323 | -0.401 | -0.385 | 0.035 |
| 15q26.1 | 0.698 | 0.486 | -0.212 | -0.593 | -0.531 | 0.147 |
| 16p13.3 | 0.849 | 0.823 | -0.026 | 1.570 | 1.637 | 0.094 |
| 17q11.2 | 0.832 | 0.804 | -0.028 | 1.029 | 0.658 | -0.772 |
| 17q25.3 | 0.798 | 0.632 | -0.166 | -0.364 | -0.475 | -0.176 |
| 19p13.13 | 0.588 | 0.556 | -0.032 | 1.028 | 1.027 | -0.023 |
| 19p13.3 | 0.670 | 0.649 | -0.020 | 1.163 | 1.043 | -0.302 |
| 19q13.41 | 0.633 | 0.464 | -0.170 | 1.193 | 1.136 | -0.192 |
| 19q13.42 | 0.712 | 0.441 | -0.270 | -0.472 | -0.405 | 0.127 |
| 20q13.33 | 0.851 | 0.834 | -0.017 | -0.317 | -0.423 | -0.218 |
| 22q13.31 | 0.811 | 0.815 | 0.004 | 1.201 | 1.126 | -0.173 |
| Xp11.23 | 0.825 | 0.799 | -0.026 | -0.165 | -0.050 | 0.292 |
| Xq13.2 | 0.861 | 0.850 | -0.011 | -0.184 | -0.061 | 0.248 |
| Xq23 | 0.687 | 0.433 | -0.254 | 0.198 | 0.224 | 0.034 |
| Xq26.2 | 0.813 | 0.558 | -0.255 | 0.606 | 0.645 | 0.025 |
| Xq26.3 | 0.661 | 0.480 | -0.180 | -0.111 | -0.273 | -0.293 |
| Xq27.3 | 0.700 | 0.448 | -0.252 | -0.579 | -0.569 | 0.013 |
| Xq28 | 0.629 | 0.329 | -0.301 | -0.744 | -0.618 | 0.261 |
| *Expression difference was z-transformed after obtaining log-ratio between background and tumor. Thus, it was not equal to simple subtraction of background from tumor. | | | | | | |

Supplementary Table S4.

A linear mixed model for miRNA cluster expression (average within each cluster) change attributed to average cluster methylation change.

|  |  |  | 95% confidence interval | |
| --- | --- | --- | --- | --- |
| Parameter | Estimate | P value | Lower | Upper |
| Intercept | 0.346 |  | 0.097 | 0.596 |
| miRNA methylation change | 1.070 | <0.001 | 0.703 | 1.437 |
| miRNA methylation in background | -0.213 | 0.200 | -0.539 | 0.113 |
| miRNA expression in background | -0.445 | <0.001 | -0.514 | -0.376 |
| Random effect: individual ID, and expression data were z, and log-transformed.  Dependent variable: miRNA expression change (background - tumor), z, and log-transformed | | | | |

Supplementary Table S5.

A linear mixed model for general protein-coding gene expression change between tumor and background attributed to their methylation change stratified by distance from CpG island and transcription start site.

|  |  |  |  |  | 95% confidence interval | |
| --- | --- | --- | --- | --- | --- | --- |
| CpG status | Location | Parameter | Estimate | P value | Lower | Upper |
| CpG island | Gene Body | Intercept | 0.060 |  | 0.050 | 0.070 |
|  |  | miRNA methylation change | 0.123 | <0.001 | 0.106 | 0.139 |
|  |  | miRNA methylation in background | -0.077 | <0.001 | -0.084 | -0.070 |
|  |  | miRNA expression in background | -0.083 | <0.001 | -0.085 | -0.080 |
|  | Promoter | Intercept | 0.211 |  | 0.170 | 0.253 |
|  |  | miRNA methylation change | -0.911 | <0.001 | -0.924 | -0.897 |
|  |  | miRNA methylation in background | -0.604 | <0.001 | -0.613 | -0.596 |
|  |  | miRNA expression in background | -0.133 | <0.001 | -0.134 | -0.131 |
| Shore | Gene Body | Intercept | 0.077 |  | 0.063 | 0.090 |
|  |  | miRNA methylation change | 0.382 | <0.001 | 0.366 | 0.399 |
|  |  | miRNA methylation in background | 0.005 | 0.183 | -0.002 | 0.013 |
|  |  | miRNA expression in background | -0.091 | <0.001 | -0.093 | -0.088 |
|  | Promoter | Intercept | 0.152 |  | 0.129 | 0.175 |
|  |  | miRNA methylation change | -0.093 | <0.001 | -0.108 | -0.079 |
|  |  | miRNA methylation in background | -0.192 | <0.001 | -0.199 | -0.186 |
|  |  | miRNA expression in background | -0.102 | <0.001 | -0.104 | -0.100 |
| Shelf | Gene Body | Intercept | -0.107 |  | -0.136 | -0.079 |
|  |  | miRNA methylation change | 1.015 | <0.001 | 0.994 | 1.037 |
|  |  | miRNA methylation in background | 0.361 | <0.001 | 0.343 | 0.378 |
|  |  | miRNA expression in background | -0.126 | <0.001 | -0.130 | -0.123 |
|  | Promoter | Intercept | -0.109 |  | -0.148 | -0.070 |
|  |  | miRNA methylation change | 0.630 | <0.001 | 0.592 | 0.668 |
|  |  | miRNA methylation in background | 0.108 | <0.001 | 0.084 | 0.132 |
|  |  | miRNA expression in background | -0.199 | <0.001 | -0.206 | -0.193 |
| Non-CpG island | Gene Body | Intercept | -0.176 |  | -0.212 | -0.140 |
|  |  | miRNA methylation change | 1.074 | <0.001 | 1.063 | 1.085 |
|  |  | miRNA methylation in background | 0.275 | <0.001 | 0.266 | 0.285 |
|  |  | miRNA expression in background | -0.186 | <0.001 | -0.188 | -0.184 |
|  | Promoter | Intercept | -0.053 |  | -0.124 | 0.018 |
|  |  | miRNA methylation change | 0.343 | <0.001 | 0.328 | 0.357 |
|  |  | miRNA methylation in background | -0.244 | <0.001 | -0.255 | -0.234 |
|  |  | miRNA expression in background | -0.188 | <0.001 | -0.191 | -0.185 |
| Random effect: individual ID, and expression data were z, and log-transformed.  Dependent variable: gene expression change (background - tumor), z, and log-transformed | | | | |  |  |
|  |  |

Supplementary Table S6.

| Context score++ |  |  |  | 95% confidence interval | |
| --- | --- | --- | --- | --- | --- |
|  | Parameter* | Estimate* | P value | Lower | Upper |
| < -0.6  (highly matched) | Intercept | 0.197 | <0.001 | 0.119 | 0.275 |
|  | miRNA methylation in background† | -0.055 | 0.047 | -0.110 | -0.001 |
|  | miRNA methylation change† | -0.266 | <0.001 | -0.363 | -0.169 |
|  | miRNA expression in background† | -0.010 | 0.102 | -0.022 | 0.002 |
|  | miRNA expression change† | -0.034 | <0.001 | -0.048 | -0.020 |
|  | Target expression in background | -0.089 | <0.001 | -0.103 | -0.074 |
|  | Target methylation in background - Gene body | 0.164 | <0.001 | 0.104 | 0.224 |
|  | Target methylation in background - Promoter | 1.024 | <0.001 | 0.892 | 1.156 |
|  | Target methylation change - Gene body | -0.573 | <0.001 | -0.644 | -0.501 |
|  | Target methylation change - Promoter | -0.665 | <0.001 | -0.825 | -0.505 |
| -0.4 to -0.6  (high-intermediately matched) | Intercept | 0.060 | 0.048 | 0.001 | 0.119 |
|  | miRNA methylation in background† | 0.018 | 0.066 | -0.001 | 0.038 |
|  | miRNA methylation change† | -0.061 | 0.001 | -0.095 | -0.026 |
|  | miRNA expression in background† | -0.007 | 0.001 | -0.011 | -0.003 |
|  | miRNA expression change† | -0.012 | <0.001 | -0.017 | -0.007 |
|  | Target expression in background | -0.181 | <0.001 | -0.186 | -0.175 |
|  | Target methylation in background – Gene body | 0.344 | <0.001 | 0.321 | 0.366 |
|  | Target methylation in background – Promoter | 1.384 | <0.001 | 1.334 | 1.435 |
|  | Target methylation change – Gene body | -0.698 | <0.001 | -0.725 | -0.670 |
|  | Target methylation change – Promoter | -1.348 | <0.001 | -1.412 | -1.285 |
| -0.2 to -0.4  (intermediately matched) | Intercept | 0.036 | 0.088 | -0.006 | 0.077 |
|  | miRNA methylation in background† | 0.015 | 0.001 | 0.006 | 0.024 |
|  | miRNA methylation change† | 0.011 | 0.130 | -0.003 | 0.025 |
|  | miRNA expression in background† | 0.003 | 0.001 | 0.001 | 0.005 |
|  | miRNA expression change† | -0.001 | 0.218 | -0.003 | 0.001 |
|  | Target expression in background | -0.195 | <0.001 | -0.197 | -0.193 |
|  | Target methylation in background - Gene body | 0.308 | <0.001 | 0.298 | 0.318 |
|  | Target methylation in background - Promoter | 1.642 | <0.001 | 1.622 | 1.663 |
|  | Target methylation change - Gene body | -0.559 | <0.001 | -0.571 | -0.547 |
|  | Target methylation change - Promoter | -1.170 | <0.001 | -1.197 | -1.143 |
| < -0.6  (highly matched)  Targeted by clustered miRNAs only | Intercept | 0.254 | <0.001 | 0.120 | 0.387 |
|  | miRNA methylation in background† | -0.132 | 0.040 | -0.259 | -0.006 |
|  | miRNA methylation change† | -0.324 | <0.001 | -0.477 | -0.171 |
|  | miRNA expression in background† | -0.019 | 0.052 | -0.038 | 0.000 |
|  | miRNA expression change† | -0.037 | <0.001 | -0.058 | -0.017 |
|  | Target expression in background | -0.073 | <0.001 | -0.096 | -0.049 |
|  | Target methylation in background - Gene body | 0.189 | <0.001 | 0.089 | 0.288 |
|  | Target methylation in background - Promoter | -0.820 | <0.001 | -0.940 | -0.700 |
|  | Target methylation change - Gene body | 0.757 | <0.001 | 0.526 | 0.988 |
|  | Target methylation change - Promoter | -0.788 | <0.001 | -1.059 | -0.517 |
| Dependent variable: average target gene expression change (background - tumor), z, and log-transformed  *Expression data were z, and log-transformed.  †Values for miRNA are average of all annotated miRNAs in each target gene transcripts. | | | | |  |
|  |  |  |  |  |  |

A linear mixed model for average target gene expression change between tumor and background attributed to corresponding miRNA methylation change.
