# Supplementary figures and images for: Global, cancer-specific microRNA cluster hypomethylation was functionally associated with the development of non-B non-C hepatocellular carcinoma
Source: Mol Cancer. 2016 Apr 30;15:31. doi: 10.1186/s12943-016-0514-6 (PMC4852433; doi:10.1186/s12943-016-0514-6)

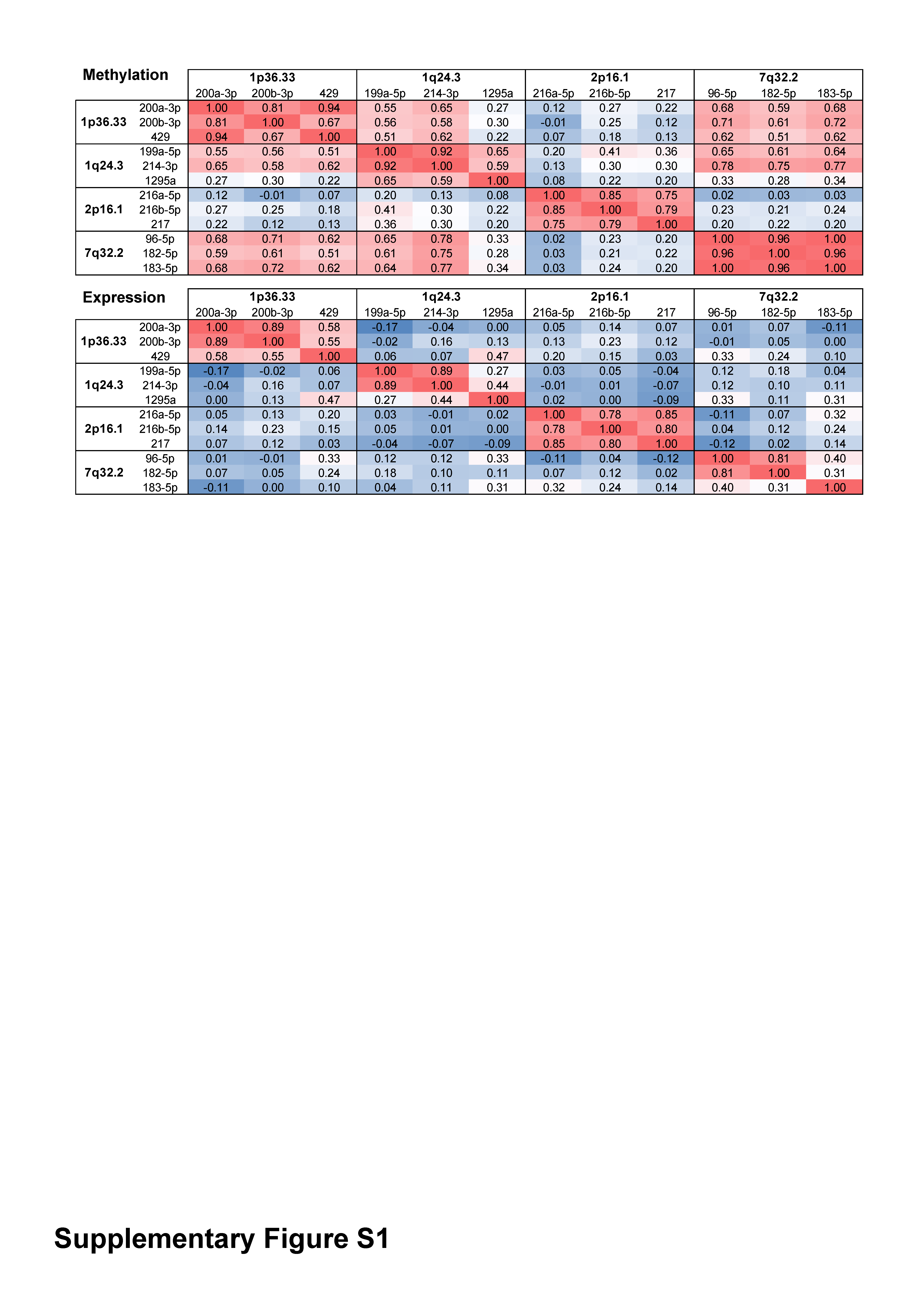

Supplement: Additional file 2: Figure S1. — Correlation coefficients between methylation/expression change of microRNAs within selected microRNA clusters (1p36.31, 1q24.3, 2p16.1, and 7q32.2). The selected clusters consist of the most and 2nd most up-/down- regulated clusters (see Supplementary Table S3). (TIFF 3351 kb) [file 12943_2016_514_MOESM2_ESM.tiff]

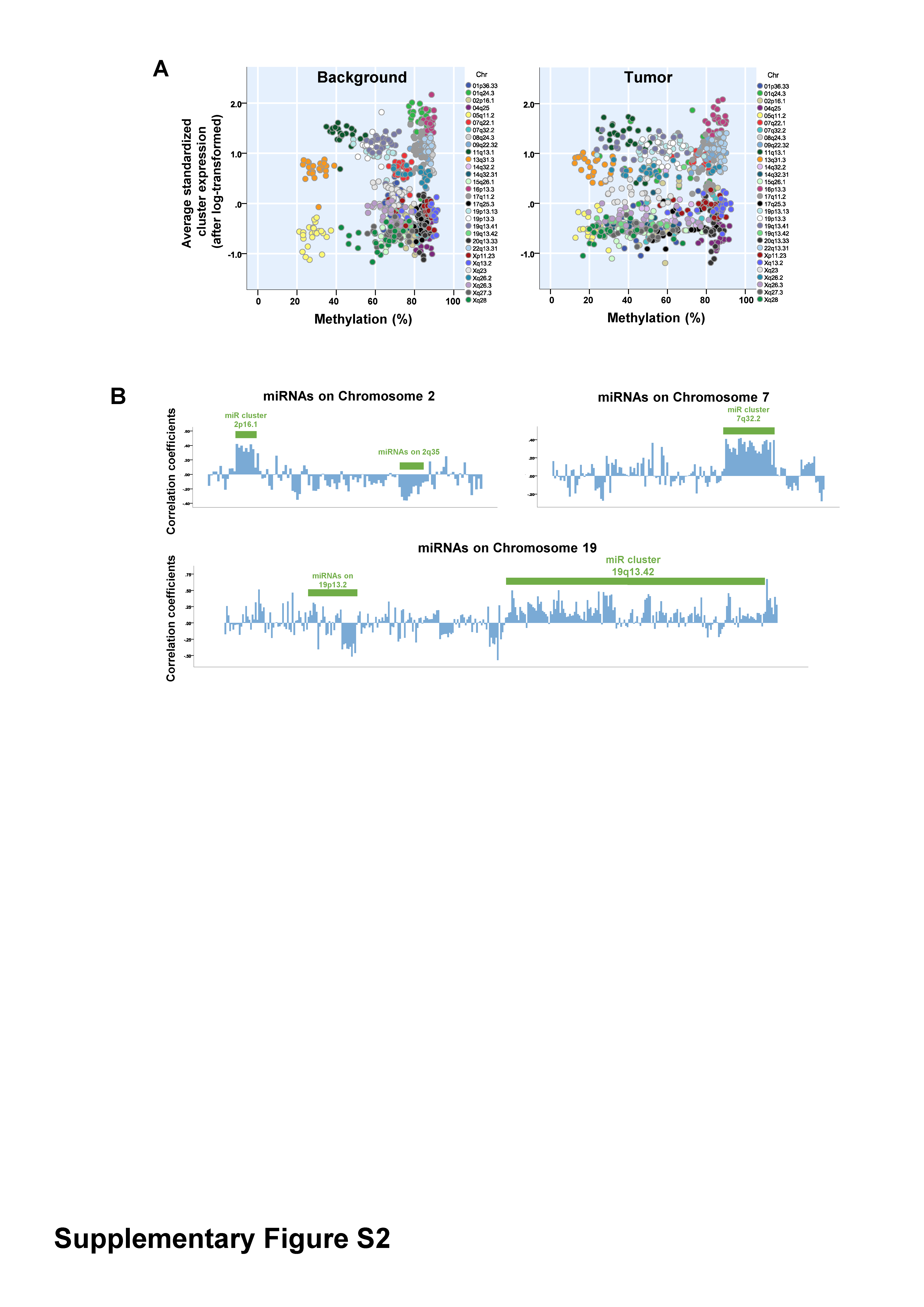

Supplement: Additional file 3: Figure S2. — Association between methylation levels of microRNA-coding regions and microRNA expression. A. Scatter plots showing correlation between clustered microRNA expression and methylation stratified by background and tumor tissues. B. Chromosome-wide correlation coefficients between methylation level as determined by each probe and corresponding microRNA expression. (TIFF 2122 kb) [file 12943_2016_514_MOESM3_ESM.tiff]
